# Usefulness of Two Types of Pain Monitors in Newborns Treated in NICU, in The Opinion of Experts: Results of The Survey

**DOI:** 10.34763/jmotherandchild.20212502.d-21-00018

**Published:** 2022-04-01

**Authors:** Wojciech Walas, Julita Latka-Grot, Tomasz Szczapa, Iwona Maroszyńska, Magdalena Rutkowska, Alicja Bartkowska-Śniatkowska, Andrzej Piotrowski

**Affiliations:** 1 Institute of Medical Sciences, University of Opole, Opole, Poland; 2 Paediatric and Neonatal Intensive Care Unit, University Hospital in Opole, Opole, Poland; 3 Department of Neonatology, Children’s Memorial Health Institute, Warszawa, Poland; 4 Department of Neonatology, Neonatal Biophysical Monitoring and Cardiopulmonary Therapies Research Unit, Poznań University of Medical Sciences, Poznań, Poland; 5 Department of Intensive Care and Congenital Malformations of Newborns and Infants, Polish Mother’s Memorial Hospital Research Institute in Łódź, Łódź, Poland; 6 Clinic of Neonatology and Intensive Care (Department of Neonatology), Institute of Mother and Child, Warszawa, Poland; 7 Department of Paediatric Anaesthesiology and Intensive Therapy, Poznań University of Medical Sciences, Poznań, Poland.; 8 Department of Anaesthesiology and Intensive Care, Children’s Memorial Health Institute, Warszawa, Poland.

**Keywords:** Pain assessment, newborn, infant, NIPE monitor, SCA monitor, survey research

## Abstract

Pain experienced in the neonatal period has been shown to have serious short- and long-term consequences. It is also known that painkillers have side effects and should not be abused. The basis of proper pain management is assessment of pain, which in newborns is very difficult due to the lack of verbal communication. In these patients, behavioural scales are used to assess pain, but they have numerous shortcomings. For this reason, many newborns treated in the ICU are at risk of pain, so instrumental methods of detecting and assessing the severity of pain are being sought.

During three months, seven Polish NICUs conducted research with the use of NIPE and SCA monitors. After this time, the heads of these departments filled in questionnaires regarding their individual opinions on the usefulness of these devices.

All respondents found pain monitors useful in the NICU. The NIPE monitor was rated slightly higher, as its usefulness in assessing the effectiveness of analgosedation and in the management of patients in the postoperative period was better rated. The high acceptance of both devices by legal guardians of newborns is noteworthy.

It should be stated that in newborns, any way to improve pain monitoring is valuable. In the opinion of Polish experts, pain monitors are useful in NICU. The NIPE monitor was assessed a little higher and was considered useful in the assessment of analgosedation and in postoperative treatment. Pain monitors can provide valuable support for pain assessment in newborns treated in the NICU.

## Introduction

Until the second half of the last century, it was believed that newborns were unable to feel pain; they thus underwent surgery without anaesthesia. Only in the mid-1980s it was proved that newborns who had anaesthesia during surgery had better short- and long-term treatment outcomes than those who were treated only with muscle relaxation [1]. Both short- and long-term negative consequences of pain experienced in the neonatal period have been demonstrated, concerning brain development, the functioning of the hypothalamic-pituitary-adrenal axis, hypersensitivity to pain stimuli, as well as cognitive and motor development disorders [2, 3, 4, 5, 6, 7, 8, 9]. This indicates the need for effective pain management in these patients; however, painkillers, especially opioids, can also cause complications, both early and late, although research results on this topic area are inconclusive [10, 11, 12, 13, 14, 15]. For these reasons, it is necessary to individually determine the indications for their use in newborns. The basis of rational pain treatment is the correct assessment of its occurrence and intensity. Over 25 years ago, it was proposed that pain should be treated as the ‘fifth vital sign’. As such, it should be monitored and documented in addition to parameters such as temperature, heart rate, blood pressure, and respiration [16,17]. However, the assessment of pain in newborns and infants is difficult due to the lack of verbal communication. It is based on behavioural or behavioural-physiological scales. These scales use the assessment of behaviour and selected life parameters. Although the usefulness of the scales has been proven, they have shortcomings, the most important of which are as follows: they are time-consuming; they require appropriately trained personnel (mainly nurses); they are subjective in the assessment of behavioural parameters; and there is lack of continuity of assessment, which may result in overlooking pain incidents [18,19].

As a result, despite the great progress made in this area in recent years, pain management in newborns is still insufficient [20, 21, 22, 23, 24]. Instrumental methods of pain assessment are an interesting support. Among them, methods based on the analysis of heart rate variability (HRV) and electrical skin conductance (SC) have been applied in newborns. The device that uses HRV is the NIPE monitor (Newborn Infant Pain Evaluation, Mdoloris Medical Systems, Loos, France), which uses an analog link to download the electrocardiography record from a standard cardiac monitor without contacting the patient directly. As a result of the analysis of respiratory HRV using complex mathematical algorithms, two indices are obtained – NIPEi and NIPEm, corresponding to the tension of the parasympathetic system. NIPEi is an instantaneous value and is primarily suitable for assessing procedural pain, and NIPEm is an average value that is mainly useful for assessing anaesthesia or analgosedation. Both indices take values from 1 to 100. According to the manufacturer's recommendation, the value of 50 is the pain threshold, so values ranging from 50 to 70 correspond to comfort, and nociceptive stimuli cause the index to fall below 50 [25, 26, 27]. The SCA monitor (Skin Conductance Algesimeter, SCA MedStorm Innovation, Norway) uses SC changes as a result of emotional sweat secretion with stimulation of the sympathetic nervous system. Three self-adhesive and atraumatic electrodes are placed on the skin of the sole of the foot of newborns and infants. The device measures and displays several parameters, of which, according to the manufacturer's recommendation, PPS (PPS – Peaks Per Second) is suitable for measuring the intensity of pain in newborns and infants. PPS levels are displayed on the monitor in five colours corresponding to different pain severity [28,29].

The aim of these studies was to find out the opinions of experts on the usefulness of instrumental pain monitoring and their subjective assessments regarding the use of the two types of monitors in neonates treated in the intensive care unit.

## Material and methods

In the period 1.11.2018 – 31.01.2019, the studies that analysed the efficacy of NIPE and SC monitors for pain assessment were carried out in in seven centres in Poland – four NICU (neonatal intensive care units) and three paediatric anaesthesiology and intensive care units. The acceptance of the local ethical committee was granted. Study was approved by the local bioethics committee.

(270: 11.10.2018). Additionally, after three months of using both devices, a questionnaire study was conducted on the subjective opinions of experts on their practical usefulness. The heads of centres were asked to complete a questionnaire on the usefulness of instrumental pain monitoring, with the following answers to choose from: 1. definitely useful, 2. rather useful, 3. rather unhelpful, 4. definitely unhelpful, 5. I have no opinion. They were also asked to score, on a scale of 1 to 5, the following eight aspects of the use of both monitors: 1. suitability for the assessment of procedural pain, 2. suitability for monitoring the effectiveness of analgosedation, 3. suitability for patient management in the postoperative period, 4. approval from parents / legal guardians, 5. acceptance by nursing staff, 6. non-invasive measurement, 7. ease of use, 8. legibility of measurement results and ease of interpretation. For each monitor the sum of the points available in seven centres for each aspect was 35, and the total score available in seven centres was 280. The study was anonymous. At the time of filling in the questionnaires, the respondents did not know the results of the conducted scientific research in those seven centres.

## Results

All respondents answered the question about the usefulness of instrumental pain monitoring in the studied group of patients. It was assessed as definitely useful in three centres and rather useful in four. All respondents scored both monitors in eight aspects. A summary of the scores for both devices is presented in Table 1.

**Table 1:** Summary of the scores for both devices in seven medical centres.

Assessed aspect	sum of points in seven centres	
	NIPE	SCA
Suitability for the assessment of procedural pain	25	24
Suitability for monitoring the effectiveness of analgosedation	31	27
Suitability for patient management in the postoperative period	32	22
Acceptance by parents / legal guardians	33	32
Acceptance by nursing staff	32	23
Non-invasive measurement	34	29
Ease of use	34	24
Readability of measurement results and ease of interpretation	29	24
Total points	250	205

## Discussion

All our respondents expressed the opinion that instrumental pain monitoring is definitely useful (4 respondents) or rather useful (3 respondents). Overall, the NIPE monitor received a higher score. The assessment of the usefulness of both devices in monitoring of procedural pain was similar and not very high. The usefulness of both monitors to assess the effectiveness of analgosedation was rated higher, while the NIPE monitor obtained a higher score than the SCA monitor. Similarly high was the rating of the NIPE monitor in terms of its usefulness in the postoperative period; this rating was also higher than the rating of the SCA monitor. The high level of acceptance by parents / legal guardians, which is very similar for both monitors, is noticeable. This is probably due to the non-invasive measurements made by NIPE and the low invasiveness of monitoring in the case of SCA monitor (the presence of additional electrodes on the newborn’s foot did not cause problems).

On the other hand, the NIPE monitor obtained a higher grade of acceptance by nurses due to non-invasiveness and lack of additional electrodes. In terms of the readability of the results and the ease of their interpretation, also the NIPE monitor was scored higher. This may be due to the ability to track both NIPE indices, to present a record with a time-varying curve, and to set thresholds for the desired values. The SCA monitor, on the other hand, offers the presentation of the measured values in colours, but their interpretation is unclear from a clinical point of view, and this monitor is also highly sensitive to artifacts. We did not find data on the subjective opinions of users on the usefulness of both monitors, and the results of scientific research on this topic are inconclusive. A significant amount of research has been done showing that the parameters measured by both devices change in response to pain stimuli, but there are very few publications regarding their practical utility in these patients in a clinical setting. Verweij et al. assessed the usefulness of NIPE in newborns after surgery performed under general anaesthesia and found that, although NIPE detects pain and discomfort in these patients, it does not correlate with the assessment using behavioural scales [30]. Gendras et al. and Cremillieux et al. found no significant correlation between the NIPE index and the PIPP-R score when performing painful procedures in preterm infants [31,32]. On the other hand, Walas et al., in a similar group of patients, found a significant decrease in this index three minutes after the stimulus. These measures correlated with the behavioural scale score, and the decline in the NIPE index occurred faster the stronger the behavioural response to pain was [33]. Similar results were obtained by Faye et al., who found a significant correlation between the NIPE index and the assessment of pain on the EDIN (Échelle Douleur Inconfort Nouveau-Né) scale, and Buyuktiryaki et al., who showed a correlation between the NIPE index and the assessment of pain intensity on this scale after the introduction of drainage of the pleural cavity in preterm newborns [27,34]. The results of studies on the usefulness of SCA in the assessment of pain in neonates are also inconclusive. In this group of patients, De Jesus et al., Pereira-da-Silva et al. and Eriksson et al. compared the results of SC measurements with the score on behavioural scales

in response to heel prick and found no correlation between them, although they noted increases in SC [35, 36, 37]. Storm described changes in SC caused by pain stimuli in preterm infants, but also found no correlation between them and the 4-point behavioural scale [29]. On the other hand, Tristão et al. observed in neonates a correlation between the increase in SC level and the modified COMFORT score in response to heel prick in infants, and Dadal et al. observed a correlation between the SC level and the NFSC score in postoperative infants [38,39].

In summary, it should be stated that in newborns, any way to improve pain monitoring is valuable. In the opinion of Polish experts, pain monitors are useful in NICU. The NIPE monitor was assessed a little higher and was considered useful in the assessment of analgosedation and in postoperative treatment. Pain monitors can provide valuable support for better care of newborns treated in the NICU.
